# Monitoring of odors emitted from stabilized dewatered sludge subjected to aging using proton transfer reaction–mass spectrometry

**DOI:** 10.1007/s11356-018-4041-4

**Published:** 2019-01-04

**Authors:** Hubert Byliński, Radosław J. Barczak, Jacek Gębicki, Jacek Namieśnik

**Affiliations:** 1 Faculty of Chemistry, Department of Analytical Chemistry, Gdańsk University of Technology, Narutowicza 11/12 Street, 80-233 Gdańsk, Poland; 2 Faculty of Building Services, Hydro and Environmental Engineering, Warsaw University of Technology, 20 Nowowiejska Street, 00-653 Warsaw, Poland; 3 UNSW Water Research Centre, School of Civil and Environmental Engineering, UNSW, Sydney, NSW 2052 Australia; 4 Faculty of Chemistry, Department of Process Engineering and Chemical Technology, Gdańsk University of Technology, Narutowicza 11/12 Street, Gdańsk, Poland

**Keywords:** Flux hood chamber, Odors, Proton transfer reaction–mass spectrometry, Sludge, Wastewater treatment plants

## Abstract

One of the potential emission sources of odorous compounds from wastewater treatment plants is sludge processing. The odorous compounds released from dewatered sludge can result in odor nuisance. This study concerns the use of flux hood chamber combined with proton transfer reaction—time of flight—mass spectrometry (PTR-MS) technique for periodical monitoring of odorous compounds emitted from aged, stabilized dewatered sludge samples from 2 different wastewater treatment plants located in Pomeranian Voivodeship, Poland. Based on determined concentration of the chemical compounds and olfactory threshold values, theoretical odor concentrations (known also as “odor activity value” or “odor index”) were calculated for 17 selected odorous compounds. As a result, sulfur compounds such as diethyl sulphide, dimethyl sulphide, methanethiol, and ethanethiol were estimated as the most significant chemical compounds responsible for malodorous effect (average results, e.g., methanethiol, 178 ou/m^3^; diethyl sulphide, 184 ou/m^3^). Based on Pearson correlation coefficient, we revealed a correlation between odorous substances emitted from aged, stabilized dewatered sludge cakes. It was revealed that stabilized dewatered sludge still possessed significant amount of odorous compounds and applied measurement technique could be used for monitoring of odor concentration level of selected malodorous compounds.

## Introduction

Wastewater treatment plants (WWTPs) are one of the significant exemplifications of human activity, which have dominant impact on air quality, especially in the areas close to emission sources (Burlingame et al. 2004; Carrera-Chapela et al. 2014). They are a complex network of technological systems, consisting of many treatment stages, with different process conditions (Xu et al. 2014). Volatile organic compounds emitted from WWTPs, including odorous compounds, can have negative effect on people, animal, and plant ecosystem (Carrera-Chapela et al. 2016a; Gębicki 2016; Byliński et al. 2017b). One of the main sources of odorous compounds emission in WWTPs is sludge processing (Chen et al. 2011; Roy et al. 2011). The presence of VOCs in sludge has been attributed to the degradation of organic material (Adams and Witherspoon 2003). Over 36% of distribution of odor emission from WWTPs is from sludge dewatering, drying, and thickening processes (Gębicki et al. 2016). Among many odorous chemical compounds emitted from sludge cakes, volatile sulfur compounds (VSCs) are commonly considered to be the main substances causing odor nuisance from WWTPs (Carrera-Chapela et al. 2016b; Fisher et al. 2018b). This fact is related to relatively low values of olfactory threshold concentration of these compounds as compared to other volatile compounds (e.g., methanethiol, 0.07 ppb; ethanethiol, 0.0087 ppb as compared to toluene, 457 ppb; benzaldehyde, 42 ppb; or acetonitrile, 13,000 ppb) (Nagata 2003). Emission of VSCs increases with the decrease in sludge oxygenation and increase in temperature during sludge treatment (Mrowiec et al. 2005). Sensory properties of odor mixtures can depend on their chemical composition. Even small modification in composition of the mixture can significantly change their sensory properties (Capelli et al. 2013a).

The products leaving anaerobic digesters, including digestate, which is a stock for dewatered sludge, are a source of odorants emission; it is necessary to process them in a way enabling their further utilization, for example in agriculture (Rosenfeld et al. 2001; Sharma et al. 2017). One of the most frequent operations is stabilization of sludge cakes—the stabilization process reduces organic matter, which leads to putrescibility, the overall microbe level increases, however the amount of pathogenic microbes decreases (Novak et al. 2003). This solution gains increasing popularity due to a possibility of biogas recovery, which is a source of renewable energy (Cieślik and Konieczka 2016). The process of sludge stabilization results in a decrease in the number of environmentally harmful substances, including those causing odor nuisance. Different approaches to the technical solutions in wastewater treatment plants, concerning conditions of the stabilization process, can influence on varying amount of odorous substances present in the sludge after the stabilization process (Qi et al. 2008; Kim et al. 2017). In order to decide about further utilization of stabilized sludge, it is necessary to determine a level of potential emission of the chemical compounds present in the sludge (Byliński et al. 2018). This evaluation can be carried out using mathematical models (Leyris et al. 2005; Lucernoni et al. 2016a) or via field measurements. In the latter case, direct measurements are a frequent solution, which employs devices enclosing investigated surface, which is a source of emission (Lucernoni et al. 2016b). Such approach is often used in the investigations concerning evaluation of wastewater treatments plants or landfills operation as far as analysis of emission level from particular elements of the installation is concerned. One of the advantages of direct measurements is a possibility of qualitative evaluation of emission from particular fragments of the emitting surface (so-called local emission). The direct measurements can provide emission rates from surfaces, rather than ambient emissions, which can be diffuse and difficult to monitor due to the influences of climate (Chen et al. 2017).

The devices used for direct determination of emission level of volatile odorous compounds can differ significantly because of applied construction design, shape, and dimensions of measurement chamber or conditions inside the chamber (Guillot et al. 2014). One of such solutions is flux hood chamber, the application of which to measurement of emission of volatile air pollutants was recommended by the United States Environmental Protection Agency (US EPA) (Klenbusch 1986). Operation of this chamber consists in generation of constant-composition mixture of volatile substances released from the samples under investigation. This is achieved by flushing with a stream of inert gas, for instance high purity nitrogen. Once uniform mixture is obtained, it is possible to sample the analytes using sorption tubes, bags, or other devices for gas samples collection (Hudson et al. 2009). The flux hood chamber is a device commonly used to measure emission of the odorous compounds generated in municipal wastewater treatment plants at particular stages of their operation (Hudson et al. 2008; Parker et al. 2013).

In order to identify and quantify odorous compounds present in gaseous samples, gas chromatography coupled with mass spectrometry (GC-MS) is commonly used (Nicolas et al. 2006; Bruno et al. 2007; Kosek et al. 2018). Typically in gas chromatography methods, a pre-concentration process is achieved by adsorbing volatile compounds from gaseous samples onto a suitable adsorbents. This pre-concentration step and desorption of analytes into the chromatographic column are time-consuming, which significantly elongates procedure (Byliński et al. 2017a; Woźniak et al. 2018). Although gas chromatography is the reference method for the analysis of VOCs in air samples, there is also growing need to develop new methods allowing direct, rapid, not time-consuming, non-invasive, and very sensitive monitoring of volatile compounds present in gaseous samples.

Among various available and investigated methods, there is proton transfer reaction–mass spectrometry technique (PTR-MS). This technique was development in the mid-1990s by Werner Lindinger group and it is based on the chemical ionization (Cappellin et al. 2012; Cui et al. 2016). The PTR-MS technique allows measurement of volatile analytes released directly from the investigated samples, without a need for complex operations of analytes enrichment. It is possible thanks to the fact that the volatile fraction is sucked directly into the ionization chamber. There occurs proton transfer reaction, only for the compounds, which exhibit higher affinity to proton than water. That is why presence of the main air components does not interfere with the results. Unquestionable advantage of the PTR-MS technique is a possibility of analytes measurement at very low concentration level (depending on spectrometer configuration, it is possible to achieve the detection level of ppb or even ppt *v*/*v*) (Jordan et al. 2009). PTR-MS technique is widely used in many applications: atmospheric chemistry, plant studies, food science, and medicinal applications (Hewitt et al. 2003; Biasioli et al. 2004; Tani et al. 2007; Blake et al. 2009). To the best of authors’ knowledge, up till now, the PTR-MS technique has not been used for sludge cakes.

The objective for this study was investigation of the capability of proton transfer reaction–mass spectrometry technique for periodical monitoring of concentration of volatile organic compounds emitted from aging anaerobically stabilizated sludge samples. Identification of the volatile organic compounds involved earlier application of the GC-MS technique as well as literature survey (Fisher et al. 2018a, c). This research can show changes in concentration of identified compounds, which can potentially contribute to odor nuisance from stabilized sludge cakes produced in WWTPs. This information can be useful to estimate a potential application of stabilized sludge in agricultural industry or other branches of human activity. Moreover, the PTR-MS technique allows evaluation odor activity values (OAV), describing which odorous compounds have the biggest contribution to strength of perceived odor of the entire gas mixture. Such situation enables optimization of the deodorization methods in order to reduce concentration of these compounds, which have the most significant impact on unpleasant odor generation.

## Experimental

### Site description and sampling location

**Fig. 1:**
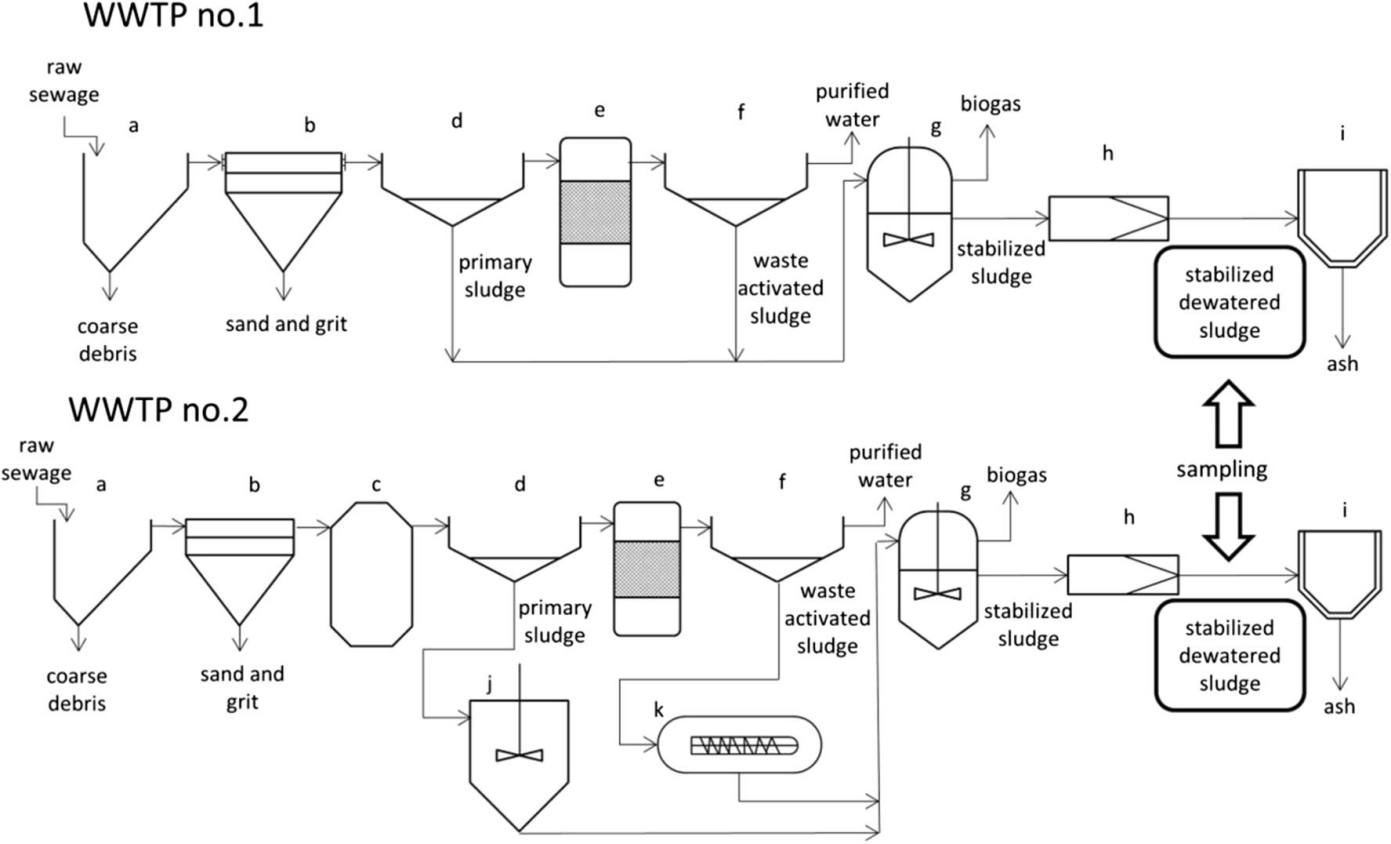
Scheme of operation in WWTPs; a-screen; b-grit chamber; c-primary reaction chamber; d-primary clarifier; e-bioreactor; f-secondary clarifier; g-digester; h-thickener; i-combustion chamber; j-radial thickener; k-thickening centrifuge

Two wastewater treatment plants located in the northern part of Poland were selected for this investigation. Similar as other WWTPs, these facilities consist of three main technological sections: mechanical, biological, and sludge treatment sections. Figure 1 presents general configuration of WWTPs operation. Both processes of sludge stabilization occur in similar technological conditions; however, in the case of WWTP no.2, an additional stage was employed. It involved densification of sludge prior to forwarding them to anaerobic-mesophilic fermentation. Table 1 contains most important information about these treatment plants.

**Table 1:** Details of wastewater treatment plants employed in the investigation

Location	WWTP no.1	WWTP no.2
Mechanical section	● 4 mechanical screens (two hook-belt screens with 6-mm clearance and two scraper screens with 10-mm clearance) ● 2 sand traps (30 m × 10.4 m × 4.5 m thickness) ● 3 primary settling tanks (diameter ca. 50 m)	● 3 hook screens (6-mm clearance) ● 1 sand trap ● 4 primary settling tanks (diameter ca. 36 m)
Biological section	6 bioreactors (summary 158,100 m^3^) and secondary settling tanks	A block of biological reactors (summary 104.000 m^3^) and 8 secondary settling tanks (diameter 42 m)
Sludge treatment	Anaerobic fermentation, temperature 37 °C, retention time 21–28 days	Anaerobic fermentation (36–38 °C, two closed fermentation chambers 5700 m^3^ each), retention time 15–20 days, external pump mixing
Thermal treatment of sludge	Dewatering of sludge using sedimentation centrifuges; incineration of sludge occurs in a furnace with sand fluidized bed (temp. 850 °C); exhaust gases are subjected to purification processes	Drying of sludge–rotary drum dryer using superheated steam as a drying agent; incineration of dried sludge occurs in a furnace with fluidized bed (temp. 850–900 °C); exhaust gases are subjected to purification processes
Amount of supplied sewage (per day)	92,200 m^3^	55,000 m^3^
Amount of waste produce during mechanical treatment (per day)	3 tons	1.5 tons
Amount of solid sludge generated (per day)	140 tons	31 tons
Amount of biogas generated during fermentation (per day)	16,500 m^3^	9500 m^3^

Stabilized dewatered sludge series were collected 6 times over a 3-month period. It means 1 series of 3 samples from each plant was collected every month on the same day. On this day, the samples were collected every 20 min. The total of 18 samples from both treatment plants were investigated. Sludge samples were collected in 20 L buckets, sealed with a lid and delivered at ambient temperature for emission analysis in the laboratory of the Gdansk University of Technology. Storage of the sludge cakes was carried out at ambient conditions (laboratory air temperature 20–25 °C).

### Instrumentation

**Fig. 2:**
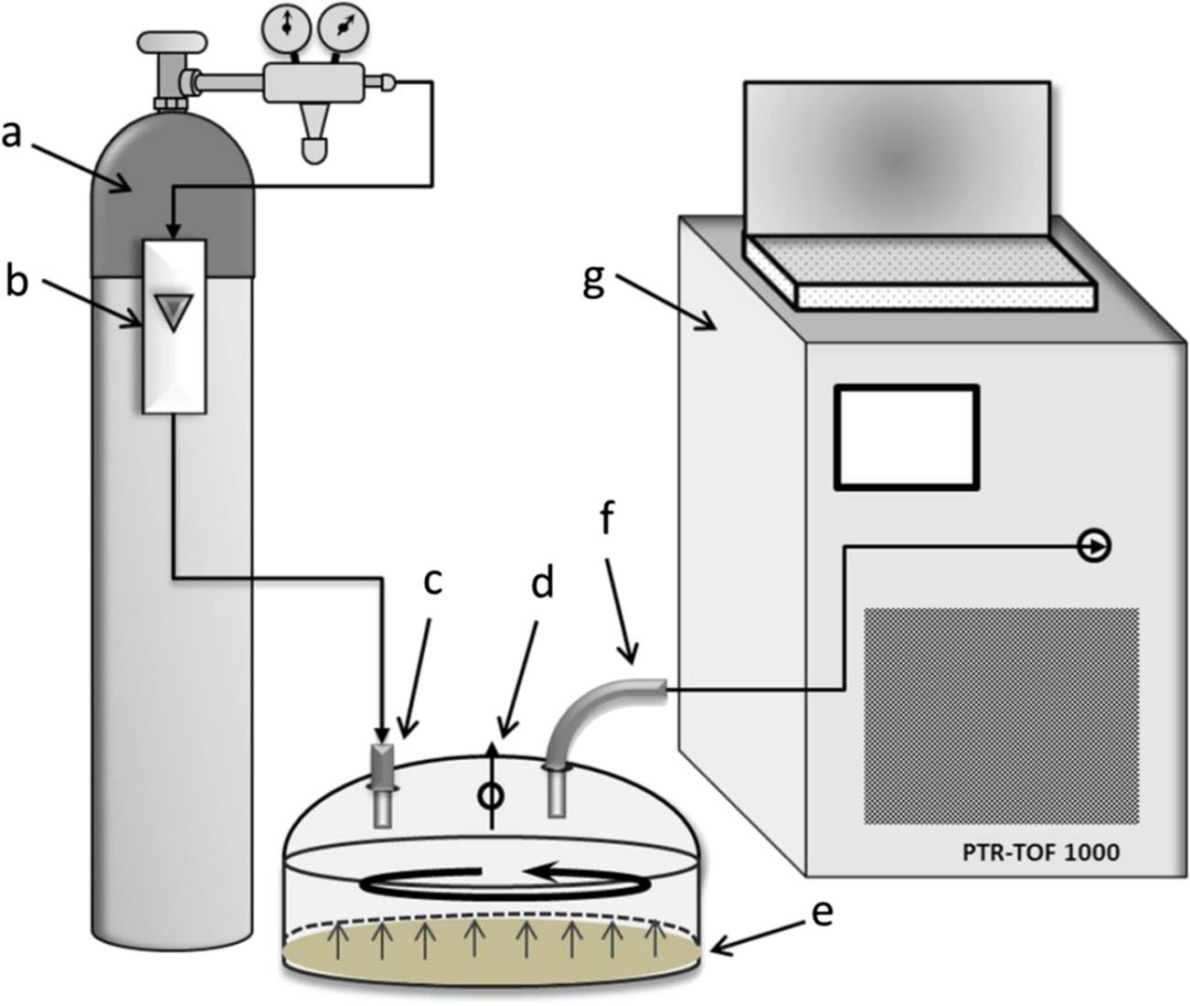
Schematic diagram of flux hood chamber combined with PTR-TOF-MS (a-carrier gas (high purity nitrogen); b-gas flow meter; c-carrier gas inlet line; d-pressure release; e-sludge surface; f-sampling line; g-PTR-TOF-MS instrument)

Determination of emission of volatile organic compounds from sludge cake samples was possible using Flux Hood Chamber combined with Proton Transfer Reaction Time Of Flight Mass Spectrometer (PTR-TOF-MS). This combination allows direct measurement of volatiles without any sample preparation. Figure 2 presents schematic diagram of flux hood chamber combined with PTR-TOF-MS.

### Flux hood chamber

The equipment consists in Plexiglas chamber (dimension 16 in.) with carrier gas inlet and outlet line. On the top of the chamber, there are small holes allowing pressure release.

During the investigation, the sludge samples were placed in buckets, in which they were transported from the treatment plants to a laboratory and stored between successive measurements. After opening the bucket, the sludge sample was enclosed in the flux hood chamber, in the way eliminating air movement between the chamber’s and the bucket’s walls (external wall of the flux hood chamber adhered to internal wall of the bucket). The chamber was mounted in this way during each measurement series. The samples were collected 3 times from every wastewater treatment plant; each time, 3 independent sludge specimens were taken for analysis. The amount of sludge collected was identical in each attempt. Before every measurement series, the sludge sample was stirred in order to ensure uniformity of the emitting surface. Prior to each measurement series, the sludge samples were purged for 30 min with a high purity nitrogen (flow rate 5 L/min, controlled using gas flow meter with no internal rubber parts).

### PTR-TOF-MS measurements

Volatile organic compounds were detected in real time using proton transfer reaction—time of flight—mass spectrometry instrument (PTR-TOF-MS 1000 Ultra, Ionicon Analytik GmbH, Innsbruck, Austria). This system allows online measurement of volatile chemical compounds in the range of ppb *v*/*v* level. During each analysis, PTR-TOF-MS transfer line (1.2-m long, inner diameter of 1 mm (PEEK tubing, BGB Analytik AG, Switzerland)) was heated to 70 °C. The drift tube was kept under controlled conditions of pressure 2.6 mbar, temperature 70 °C, mass range of *m*/*z* = 30 to *m*/*z* = 240 Da and voltage 600 V, resulting in a field density ratio (E/N) of 122 Td (E being the electric field strength and N the gas number density; 1 Td = 10^−17^ Vcm^2^). In order to record the mass spectra, IonTOF v. 2.4.40 software was used. Data processing was performed with PTR-TOF-MS Viewer v. 3.2.3.0. Tentative identification was performed based on the measured mass, isotopic ratios, and fragmentation spectra. Identified compounds were compared with the literature concerning composition of sludge samples (Fisher et al. 2017a). VOC concentration was calculated from peak areas, according to the formula described in the literature (Lindinger et al. 1998). The reaction rate coefficients used for quantification of analytes were based on the literature (Lindinger et al. 2001; Ammann et al. 2004; Zhao and Zhang 2004; Rinne et al. 2007; Taipale et al. 2008; Cappellin et al. 2012). When no data was available, the rate of *k*_R_ = 2 × 10^−9^ cm^3^/s was used. Limit of quantification was set at 10× standard deviations of the background noise recorded for a blank sample (Konieczka and Namieśnik 2007).

### Theoretical odor concentration

High concentration of particular substance present in gaseous samples does not always produce strong odor. One of the parameters, which can inform us about odor characteristic is olfactory threshold value (OT_i_). Based on OT_i_ values for all quantified substances present in odorous gas mixture and their chemical concentration values, it is possible to estimate odor activity value (OAV) of single odorous compounds (Eq. 1) and sum of theoretical odor concentrations of monitored compounds (Eq. 2):

1 $$ \mathrm{OAV}=\frac{C_{\mathrm{i}}}{{\mathrm{OT}}_{\mathrm{i}}} $$

2 $$ {C}_{\mathrm{od},\mathrm{OT}}=\sum \limits_{i=1}^n\frac{C_{\mathrm{i}}}{{\mathrm{OT}}_{\mathrm{i}}} $$

where *C*_i_ is the analytical concentration of odorous compound *i* [ppbv], OT_i_ is the olfactory threshold concentration of compound *i* [ppbv], *n* is the number of compounds in the odorous mixture, OAV is defined as the odor activity value of single odorous compounds [ou/m^3^] and *C*_od,OT_ is defined as sum of theoretical odor concentrations of monitored compounds [ou/m^3^]. Based on the ratios between measured concentrations and olfactory threshold concentrations, a simple estimation of relative contribution of odor of sludge samples, in the absence of sensory measuring techniques, can be used. It must be emphasized that the theoretical odor concentration of mixture can differ from the value of odor concentration determined with dynamic olfactometry, for instance, due to presence of odor interaction effects (Byliński et al. 2017b).

### Specific odor emission rates

Application of the flux hood chamber for measurement of emission of volatile odorous compounds, generated due to aging of sludge cakes, allows determination of a parameter known as specific odor emission rate (SOER). According to a definition, value of this parameter is a function of flow rate of the gas introduced to the measurement chamber, surface area of sludge being a source of emission and odor concentration determined for the investigated compounds (Capelli et al. 2013b). In the case of performed investigations, odor concentrations were not determined using dynamic olfactometry technique, commonly applied in this type of research. The values of odor concentration, adapted to calculation of SOER parameter, were determined instrumentally (see “Theoretical odor concentration” section), which is an alternative to the olfactometric approach and contributes to shortening of the time necessary for result acquisition. The values of SOER parameter, determined using theoretical odor concentrations of mixture, were calculated using the following equation (Eq. 3):

3 $$ \mathrm{SOER}=\frac{C_{\mathrm{od},\mathrm{OT}}\times {Q}_{\mathrm{N}}}{A} $$

where SOER, specific odor emission rate [ou/m^2^s]; *C*_od,OT_, sum of theoretical odor concentrations of monitored compounds [ou/m^3^]; *Q*_N_, flow rate of nitrogen into chamber [m^3^/s]; and *A*, surface area enclosed by the chamber [m^2^].

### Statistical analysis

Statistical analysis was carried out using SPSS Statistics software (Version 21.0, SPSS Inc., Chicago, Illinois, USA). Analysis of correlation was conducted in order to determine dependences between concentration of particular compounds emitted from stabilized sludge cakes. Determined correlations were meant to pertain to all monitored compounds as well as to particular groups of chemical compounds. Special attention was paid to the correlations occurring in case of both samples of sludge cakes. The values of Pearson correlation coefficient were determined for significance levels 95% and 99%.

## Results and discussion

### Concentration of VOCs emitted from sludge

The chemical concentrations of 17 volatile compounds emitted from aged, stabilized sludge samples from two different wastewater treatment plants are shown in Table 2.

**Table 2:** Average concentrations of tentatively identified compounds emitted from stabilized sludge cakes (*SD* standard deviations)

Tentatively identified compounds, protonated molecular formula, and protonated mass (amu)	Concentration (ppbv) ± SD									
	WWTP no.1					WWTP no. 2				
	1st day	7th day	10th day	14th day	21st day	1st day	7th day	10th day	14th day	21st day
Diethyl sulphide (DES) (C_4_H_10_S)H^+^ (91.0576)	1.84 ± 0.09	1.43 ± 0.22	1.02 ± 0.31	1.01 ± 0.04	0.75 ± 0.04	6.08 ± 0.15	2.83 ± 0.88	2.24 ± 0.18	2.02 ± 0.27	1.95 ± 0.17
Dimethyl sulphide (DMS) (C_2_H_6_S)H^+^ (63.0263)	18.67 ± 0.82	10.10 ± 0.90	11.07 ± 1.13	12.60 ± 1.13	2.43 ± 0.07	10.57 ± 9.43	12.20 ± 1.23	6.35 ± 3.81	5.23 ± 5.08	3.53 ± 1.08
Ethanethiol (ETH) (C_2_H_6_S)H^+^ (63.0263)	0.44 ± 0.07	0.20 ± 0.01	0.21 ± 0.02	0.29 ± 0.02	0.24 ± 0.07	0.82 ± 0.10	0.31 ± 0.03	0.25 ± 0.04	0.21 ± 0.03	0.20 ± 0.06
Methanethiol (MTH) (CH_4_S)H^+^ (49.0107)	10.12 ± 0.31	4.15 ± 0.18	3.4 ± 0.21	3.05 ± 0.06	2.95 ± 0.15	12.43 ± 0.53	4.88 ± 0.74	4.55 ± 0.82	3.58 ± 0.65	2.91 ± 0.22
Acetonitrile (ACN) (C_2_H_3_N)H^+^ (42.0338)	12.59 ± 0.76	12.09 ± 0.84	11.66 ± 0.83	10.82 ± 0.34	5.60 ± 0.19	5.46 ± 0.21	7.92 ± 0.10	6.65 ± 0.20	11.96 ± 1.06	13.17 ± 1.50
Pyridine (PIR) (C_5_H_5_N)H^+^ (80.0495)	1.35 ± 0.04	1.33 ± 0.40	2.81 ± 1.38	0.47 ± 0.11	1.39 ± 0.02	2.91 ± 0.21	0.53 ± 0.10	0.31 ± 0.04	0.42 ± 0.08	0.75 ± 0.28
Benzene (BEN) (C_6_H_6_)H^+^ (79.0542)	3.69 ± 0.34	3.57 ± 0.19	2.97 ± 0.35	1.69 ± 0.06	0.93 ± 0.12	10.48 ± 5.36	4.67 ± 0.70	2.13 ± 0.16	2.28 ± 0.16	3.13 ± 0.45
Toluene (TOL) (C_7_H_8_)H^+^ (93.0699)	4.46 ± 0.32	2.99 ± 0.22	2.62 ± 0.55	1.57 ± 0.07	0.92 ± 0.08	26.35 ± 0.50	11.86 ± 0.94	13.35 ± 2.23	20.77 ± 1.80	24.96 ± 3.78
Xylene* (XYL) (C_8_H_10_)H^+^ (107.0855)	7.37 ± 0.48	15.18 ± 0.47	14.56 ± 2.04	7.98 ± 0.20	1.72 ± 0.15	5.29 ± 0.11	4.29 ± 0.51	4.69 ± 0.37	5.51 ± 0.50	5.61 ± 0.67
Cymene* (CYM) (C_10_H_14_)H^+^ (135.1168)	3.29 ± 0.14	2.37 ± 0.20	2.06 ± 0.35	1.82 ± 0.13	0.94 ± 0.03	6.14 ± 0.12	7.65 ± 0.83	4.84 ± 0.26	10.78 ± 1.30	10.38 ± 1.84
1,2,3-trimethylbenzene (TMB) (C_9_H_12_)H^+^ (121.1012)	4.50 ± 0.18	3.38 ± 0.24	3.03 ± 0.47	2.02 ± 0.12	1.08 ± 0.07	5.08 ± 0.08	5.25 ± 0.60	3.95 ± 0.22	7.00 ± 0.71	7.06 ± 1.02
1-propanol (PRO) (C_3_H_8_O)H^+^ (61.0648)	34.94 ± 2.96	17.55 ± 0.90	11.05 ± 1.52	6.67 ± 0.39	5.25 ± 1.04	27.09 ± 1.24	23.52 ± 3.49	12.97 ± 1.07	12.56 ± 1.99	21.64 ± 4.75
Ethanol (ETN) (C_2_H_6_O)H^+^ (47.0491)	10.64 ± 0.18	13.86 ± 0.39	11.83 ± 1.46	10.48 ± 6.57	12.12 ± 1.33	22.19 ± 1.66	21.49 ± 1.78	18.68 ± 0.83	16.53 ± 1.24	22.29 ± 2.24
Acetaldehyde (ACA) (C_2_H_4_O)H^+^ (46.0413)	7.87 ± 0.67	7.20 ± 0.90	7.76 ± 0.57	9.58 ± 1.09	6.23 ± 0.57	27.21 ± 0.94	18.75 ± 2.24	15.83 ± 0.52	19.98 ± 1.04	23.08 ± 1.72
Benzaldehyde (BZA) (C_7_H_6_O)H^+^ (107.0491)	6.54 ± 0.38	13.81 ± 0.59	13.33 ± 1.10	7.09 ± 0.50	1.52 ± 0.19	4.80 ± 0.19	3.78 ± 0.50	4.08 ± 0.38	4.84 ± 0.60	5.27 ± 0.71
Acetone (ACT) (C_3_H_6_O)H^+^ (59.0491)	23.51 ± 1.50	9.93 ± 1.83	8.41 ± 1.05	6.12 ± 0.77	5.52 ± 0.74	37.91 ± 2.15	33.82 ± 2.64	27.72 ± 3.46	23.30 ± 1.38	33.89 ± 1.39
α-pinene (PIN) (C_10_H_16_)H^+^ (137.1325)	13.92 ± 0.67	7.65 ± 0.76	5.39 ± 0.83	3.01 ± 0.11	2.29 ± 0.13	14.05 ± 0.25	20.88 ± 1.47	13.93 ± 0.80	33.27 ± 2.91	33.13 ± 2.05

*Mixture of isomers of these compounds

Chemical compounds present in this table were selected as the main odorous compounds emitted from sludge cakes based on the available literature (Rosenfeld et al. 2001; Fisher et al. 2017a, b) and quantitative analysis of sludge samples using thermal desorption-gas chromatography coupled with mass spectrometry technique.

Composition of volatile fraction from flux hoods headspace of the sludge cakes was changing significantly during execution of the investigation. The highest concentrations, determined during the measurements conducted on the day of sludge collection from the treatment plant, were observed for:

- the sludge sample from the treatment plant no. 1 for dimethyl sulphide, 1-propanol, and acetone; they were at the level of 18–35 ppbv,
- the sludge sample from the treatment plant no. 2 for dimethyl sulphide, acetone, benzene, toluene, 1-propanol, and acetaldehyde; they were at the level of 26–40 ppbv.

In case of the sludge sample from the treatment plant no. 2, the concentrations were significantly higher for most of the monitored compounds. For example, 14 out of 17 compounds exhibited higher concentration on the first day of measurements. It was observed that concentration of some compounds decreased during the entire 21-day measurement cycle. The examples are 1-propanol and cymene for the sludge from the treatment plant no. 1 or ethanethiol and diethyl sulphide for the sludge from the treatment plant no. 2. In the other cases, it was noticed that on the seventh day since the sample collection concentration of a given compound was substantially lower than on the first day of measurements, however, the differences in concentration between the seventh and twenty-first day were not so significant (for instance acetone, methanethiol for sludge from the treatment plant no. 1 or benzene and methanethiol for the sludge from the treatment plant no. 2). In the case of some compounds, the concentrations underwent small fluctuations throughout the period of investigation (for example acetaldehyde and ethanethiol for the sludge from the treatment plant no. 1 or benzaldehyde for the sludge from the treatment plant no. 2).

Composition of volatile fraction of stabilized sludge cakes subjected to aging can differ depending on the origin of particular sludge sample. Both treatment plants also differ in selected technological parameters (see Table 1), for instance, time of sludge retention at the anaerobic stabilization stage (Lewkowska et al. 2016). Site planning around the plant can play an important role in composition of sludge. In heavy industrialized regions with the facilities lacking wastewater treatment plants, composition of wastewater can be especially rich in the compounds characteristic for an operation profile of particular facility (Fisher et al. 2017b). In the case of dominant contribution of households or residential areas, a profile of wastewater supplied to a treatment plant will be completely different (Alvarez et al. 1999). It should be emphasized that the PTR-MS technique allowed identification and measurement of ethanethiol and methanethiol, the compounds which are easily converted to sulphides upon measurements with other methods (Gruchlik et al. 2013)

### Theoretical odor concentration

**Fig. 3:**
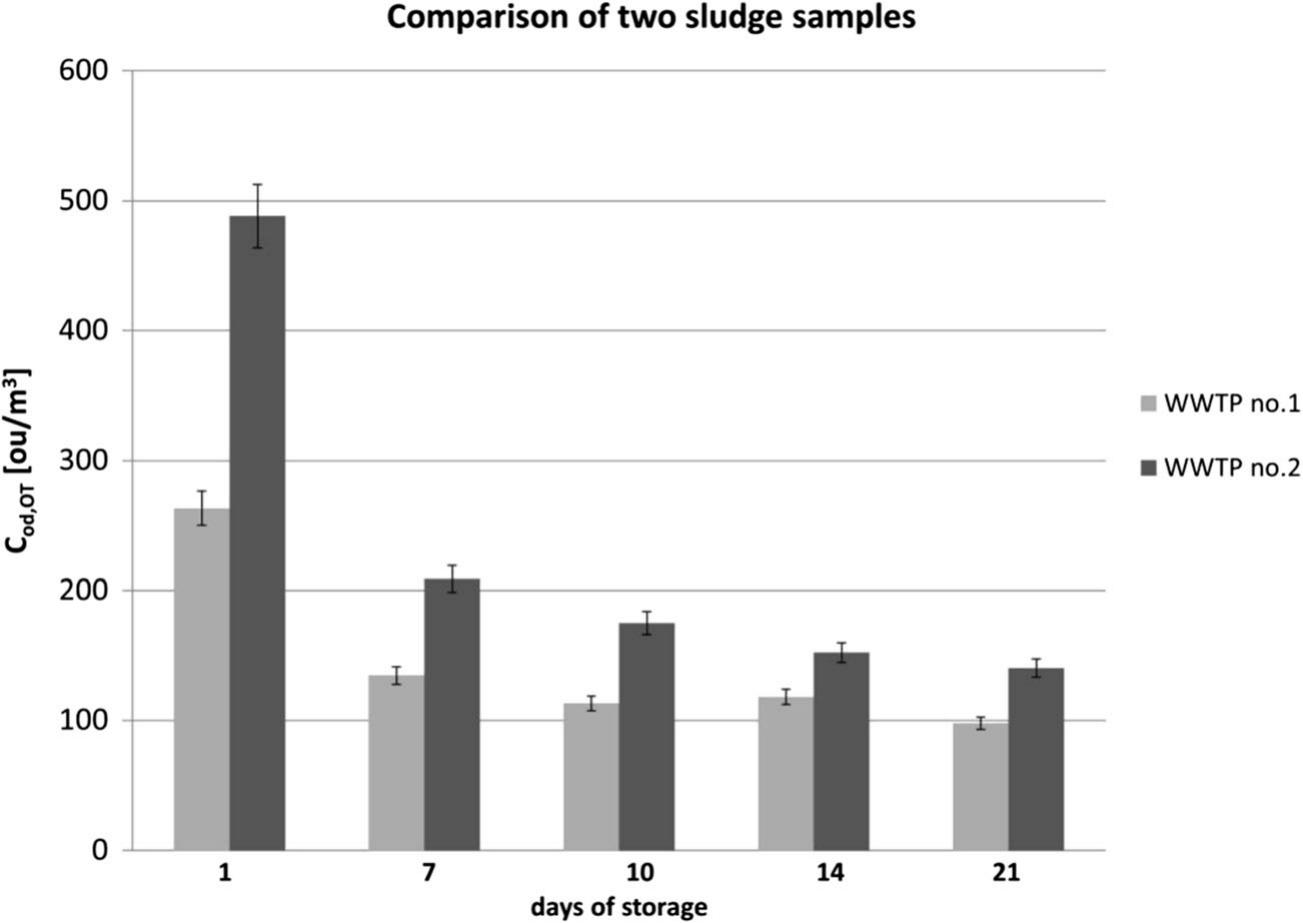
Average values of sum of theoretical odor concentrations of monitored compounds (*C*_od,OT_) estimated for 2 sludge cakes during 3 weeks

Tentatively identified compounds present in Table 2 can have different impact on odor nuisance associated with treatment of sludge cakes. These compounds have different odor properties, which also depend on composition of odorous mixture and some external factors (air temperature and humidity, intensity of solar radiation). In order to define which of these compounds have most significant impact on unpleasant smell, odor activity value for each compound (OAV) and sum of theoretical odor concentrations of monitored compounds (*C*_od,OT_) were calculated. In Table 3, an example of calculation of OAV and *C*_od,OT_ was shown. In Fig. 3, a summary of *C*_od, OT_ was compared taking into account type of WWTP and measurement day.

**Table 3:** Example of calculation of OAV (sludge sample from WWTP no 2. at 1st day of storage).* olfactory threshold was calculated as average of olfactory threshold for 3 isomers of this compound

Name of compound	Odor description	OT_i_ [ppbv] (Nagata 2003)	OAV [ou/m^3^]		
			Minimum	Maximum	Average
Diethyl sulphide	Sulfurous, onion, leek,	0.033	179.7	188.8	184.2
Methanethiol	Cheese, cooked cabbage, fishy, garlic, gasoline, meaty, rotten egg, sulfurous	0.07	170.1	185.1	177.6
Ethanethiol	Garlic-like, skunk-like, strong	0.0087	82.5	105.0	93.8
Acetaldehyde	Ethereal, fresh, fruity, pungent	1.5	17.5	18.8	18.1
Dimethyl sulphide	Cabbage, fruity, gaseous, gasoline, moldy, sulfurous, vegetable soup	3	13.5	14.2	13.9
Acetone	Characteristic, sweetish, fragrant	42,000	< 1		
Acetonitrile	Aromatic, characteristic, sweet, ethereal	13,000	< 1		
Cymene	Balsamic, citrus, fruity, fuel gasoline, herbaceous, lemon, solvent, spicy, sweet	7165^*^	< 1		
Benzene	Paint thinner	2700	< 1		
Ethanol	Alcoholic, pungent, sweet	520	< 1		
Toluene	Caramelized, ethereal, fruity, paint, pungent, rubber, solvent,	457	< 1		
Xylene	cold meat fat, plastic	380^*^	< 1		
α-pinene	Camphor, citrus, fruity, green, lime, pine, sweet, terpenic, turpentine, woody	180	< 1		
1,2,3-trimethylbenzene	Characteristic, distinctive, aromatic	120	< 1		
1-propanol	Alcoholic, fruity, musty, plastic, pungent	94	< 1		
Pyridine	Cold meat fat, fishy, rancid	63	< 1		
Benzaldehyde	Almond, burnt sugar, fruity, woody	42	< 1		
*C*_od,OT_			463.9	512.6	488.3

Based on the results presented in Table 3 and Fig. 3, it can be observed that strength of perceived odor differed for both samples, although this difference diminished with time as far as given measurement day is concerned. The highest values of sum of theoretical odor concentrations of monitored compounds were determined on the day of the samples collection from the plants. Storage and aging of the sludge resulted in a decrease in odor concentrations. It can also be noticed that the rate of sum of theoretical odor concentrations of monitored compounds decrease diminished after 10 days and stabilized as far as the samples collected from wastewater treatment plant are concerned. However, obtained values were still at the level of 100 ou/m^3^, which means significant exceeding of the admissible levels accepted in many countries with strict policy regarding emission of odorous compounds (Bokowa 2010; Van Harreveld et al. 2013; Guillot and Milan 2016).

**Fig. 4:**
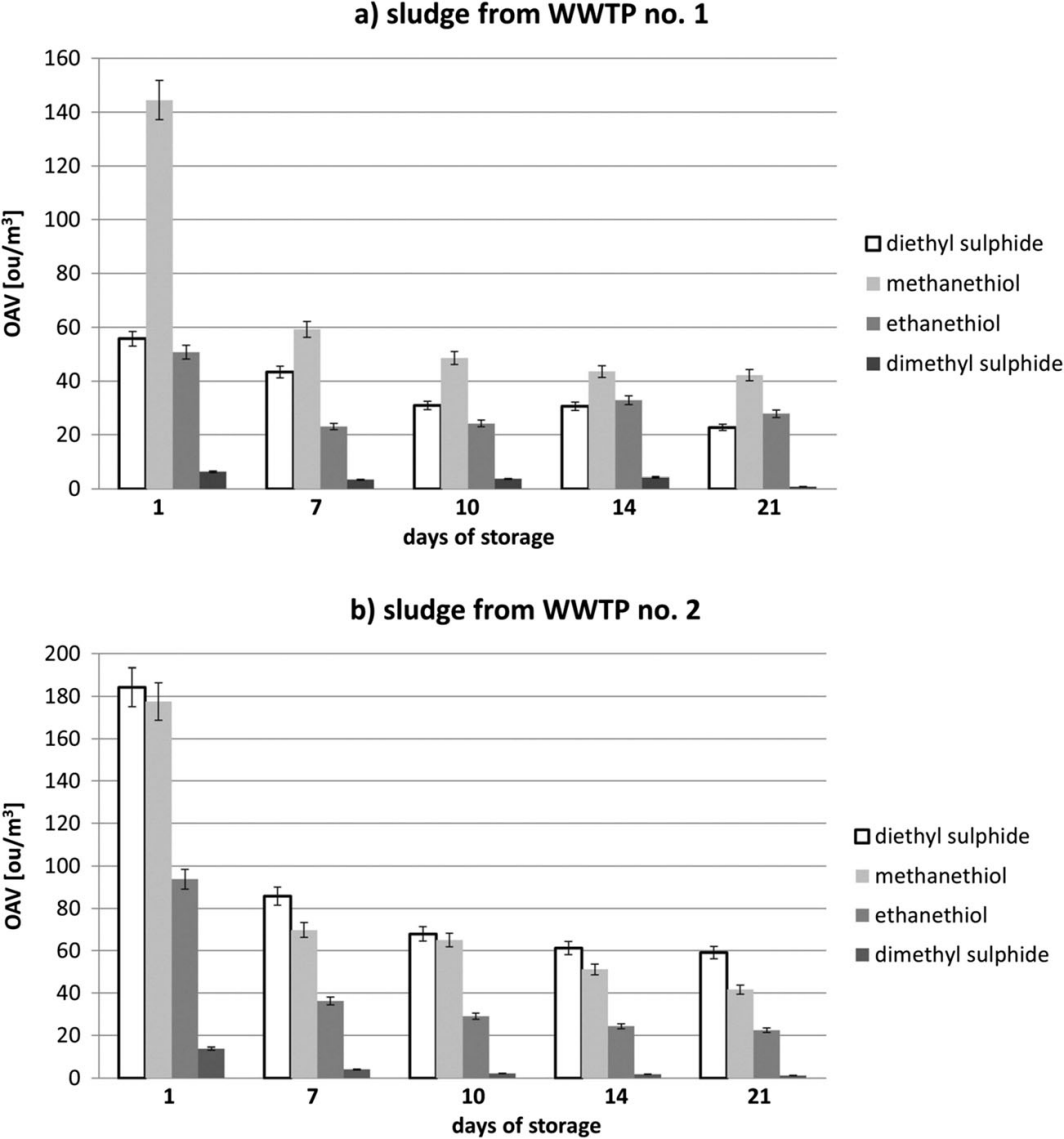
Odor activity values for 4 identified compounds; **a** sludge cake from WWTP no. 1, **b** sludge from WWTP no. 2

Comparison of the sum of theoretical odor concentrations of monitored compounds reveals that in case of every measurement day, *C*_od,OT_ values of the sludge collected from the treatment plant no. 2 were higher than the one determined for the sludge from the treatment plant no. 1. Such situation can suggest two dominant technological issues. Firstly, the wastewater treatment plant no. 1 processes bigger amount of wastewater, which leads to dilution of the entire contaminants load, thus to dilution of the odorous compounds. Secondly, duration of anaerobic fermentation in a technological process of the treatment plant no. 1 is longer, which allows transfer of bigger amount of volatile organosulphur compounds and volatile organic compounds to biogas. The data presented in Table 3, concerning the values of olfactory threshold of particular odorants and OAV determined for them, suggest that organosulphur compounds (methanethiol, ethanethiol, methyl sulphide, diethyl sulphide) had the biggest influence on summary odor concentration. These compounds are characterized by relatively low values of olfactory threshold, which implies that they can have a dominant impact on strength of perceived odor, even if they are present at low concentration level. Figure 4 illustrates changes of OAV for four aforementioned compounds during the entire period of the investigation. Analysis of these data reveals that methanethiol (in case of the treatment plants no. 1 and no. 2) and diethyl sulphide (in case of the treatment plants no. 2) exhibited the biggest influence on the level of perceived odor.

### Emission from sludge cakes

The information concerning concentration changes of particular compounds due to aging of stabilized sludge cakes and corresponding OAV odor concentrations constitutes valuable data reflecting possible changes of odor character of sludge cakes. Defining of potential influence of odorants released to the environment should also take into account the factors connected with the rate of release and propagation of the odorants.

In order to determine emission level of volatile odorous compounds, two equipment solutions are most commonly employed–flux chamber, utilized in the investigations described in this paper, and wind tunnel system. Fundamental difference between these two devices is connected with geometrical parameters and internal circulation of the gas flushing an odorant-emitting surface. Application of these solutions allows evaluation of odorants emission level defined as SOER. Table 4 gathers the values of SOER parameter obtained for the samples of sludge cakes during the investigation of odorous compounds emission as well as some literature examples determined for different potential sources of odorous compounds emission, taking into account type of the chamber used for odorants emission measurement.

**Table 4:** Specific odor emission rates for sludge samples and other literature examples

		SOER [ou/m^2^s]				
Days of storage:		1st	7th	10th	14th	21st
Stabilized biosolids samples	WWTP no. 1	0.157 ÷ 0.180	0.078 ÷ 0.094	0.062 ÷ 0.082	0.072 ÷ 0.079	0.056 ÷ 0.069
	WWTP no. 2	0.297 ÷ 0.328	0.102 ÷ 0.165	0.099 ÷ 0.125	0.073 ÷ 0.122	0.058 ÷ 0.121
Literature examples						
Sources of emission	Type of chamber		SOER [ou_E_/m^2^s]			ref.
Municipal solid waste landfills	Wind tunnel		2.1 ÷ 8.9			(Sironi et al. 2005)
	“LabOlf” sampling device (homemade)		0.011			(Lucernoni et al. 2016b)
Agricultural odor sources	Flux hood chamber		0.194 ÷ 1.973			(Hudson et al. 2009)
	Wind tunnel		13.47 ÷ 229.0			
Livestock facilities	Wind tunnel	Polypropylene cover	1.3 ÷ 2.1			(Hudson et al. 2006, 2008)
			12.2 ÷ 57.0			
	Wind tunnel	Straw cover	0.5 ÷ 4.6			
			3.3 ÷ 39.7			

Based on the emission of odorous compounds illustrated in Table 4, it can be stated that higher values were obtained for the sludge originating from the treatment plant no. 2, which is a consequence of higher values of OAV identified in the samples collected from this plant. Analogously, it was observed the value of SOER parameter also decreased upon aging of the samples.

Evaluation of emission of the odorous compounds released from different industrial and municipal sources can be an indicator of environmental impact of particular facility (Wu et al. 2017). Different values of odorous compounds emission can be obtained depending on type of odorants emission sources, methodology of investigation, and design solutions applied (geometrical dimensions, type of utilized material, circulation of gas stream inside the chamber)—selected examples are presented in Table 4. The information presented shows that magnitude of the SOER parameter is strongly diversified, both within single emission source as well as type of measurement chamber. In majority of cases, literature values are higher than these calculated for sludge cake samples. This difference can stem from a couple of factors. In the case of literature data, odorant emission level was calculated based on the odor concentrations evaluated using dynamic olfactometry technique, being a reference method in olfactometric investigations. In our studies, OAV were determined only on the basis of concentrations of particular compounds and their olfactory thresholds, which could have influenced on lower values of odor concentrations and thus on lower values of odorants emission. Instrumental investigations do not take into consideration the effect of odor interactions (for example odor synergism), but only sum of odor concentrations of particular compounds (Gębicki 2016). Moreover, the investigation engulfed only 17 odorous compounds, whereas holistic examination using dynamic olfactometry does not impose any limitation as far as the number of compounds in odorous mixture is concerned. Furthermore, in many cases, odorants emission level is determined with respect to “in situ” process, not to the material obtained as a product of selected unit process (Byliński et al. 2017a). Prior to the measurements, the sludge samples were transported to a laboratory, which could also have an influence on lower concentration of odorants determined during the analyses.

### Statistical analysis

In order to describe mutual dependences between concentrations of particular compounds present in volatile fraction of stabilized sludge cakes, the numerical values of Pearson correlation coefficient were determined and included in Table 5, together with the concentration values obtained for both treatment plants. These data indicate strong, positive correlations between some compounds belonging to the same class of chemical compounds, especially organosulphur compounds:

- Methanethiol revealed strong, positive correlation with ethanetiol and diethyl sulphide (in case of the sludge from the treatment plant no. 1 *r*^2^_I_ = 0.993, *r*^2^_I_ = 0.896, respectively, in case of the sludge from the treatment plant no. 2 *r*^2^_II_ = 0.996, *r*^2^_II_ = 0.994) and dimethyl sulphide with diethyl sulphide (*r*^2^_I_ = 0.827, *r*^2^_II_ = 1000).
- Strong, positive correlation was noticed between dimethyl sulphide and ethanethiol and methanethiol (in case of the sludge from the treatment plant no. 2 *r*^2^_II_ = 0.998 and *r*^2^_II_ = 0.994, respectively, however *r*^2^_I_ = 0.723, *r*^2^_I_ = 0.765).

**Table 5:** Pearson correlation coefficients between different odorous compounds emitted from sludge cakes (*n* = 5, 1st line concerns WWTP no. 1 and 2nd line, WWTP no. 2)

	ACN	ACA	MTH	ACT	PRO	ETH	BEN	TOL	BZA	PIN	TMB	ETN	XYL	CYM	DMS	PIR	DES
ACN	1	.534	.490	.558	.627	.292	.881^*^	.802	.746	.669	.868	.049	.756	.872	.863	.120	.747
		− .030	− .734	− .310	− .294	− .688	− .600	.281	.600	.981^**^	.904^*^	− .205	.590	.931^*^	− .689	− .504	− .691
ACA		1	.039	.040	.009	.294	.079	.100	.195	.011	.168	.832	.208	.298	.610	− .397	.137
			.684	.927^*^	.745	.737	.809	.884^*^	.654	.038	.330	.575	.584	.170	.731	.874	.734
MTH			1	.995^**^	.968^**^	*.883* ^*^	.631	.871	− .084	.950^*^	.808	− .349	− .075	.850	.765	− .051	*.896* ^*^
				.862	.631	*.996* ^**^	.982^**^	.407	.025	− .658	− .403	.409	.014	− .528	*.994* ^**^	.949^*^	*.994* ^*^
ACT				1	.985^**^	.840	.704	.916^*^	.016	.974^**^	.862	− .385	.024	.890^*^	.790	.020	.921^*^
					.617	.886^*^	.935^*^	.814	.527	− .247	.042	.428	.502	− .126	.872	.967^**^	.874
PRO					1	.753	.796	.955^*^	.141	.997^**^	.913^*^	− .417	.150	.926^*^	.784	.026	.968^**^
						.686	.698	.385	.101	− .244	− .009	.905^*^	− .046	− .109	.699	.725	.705
ETH						1	.269	.597	− .402	.711	.527	.027	− .390	.642	.723	− .329	.672
							.993^**^	.455	.073	− .610	− .344	.464	.050	− .474	*.998* ^**^	.969^**^	*.999* ^**^
BEN							1	.928^*^	.710	.837	.959^**^	− .405	.716	.903^*^	.721	.312	.858
								.559	.193	− .524	− .245	.481	.165	− .387	.988^**^	.991^**^	.990^**^
TOL								1	.415	.974^**^	.992^**^	− .407	.423	.977^**^	.838	.199	.955^*^
									.920^*^	.308	.537	.296	*.889* ^*^	.377	.432	.654	.436
BZA									1	.213	.518	− .118	*1.000* ^**^	.421	.317	.457	.284
										.591	.727	.107	.979^**^	.604	.046	.308	.051
PIN										1	*.939* ^*^	− .437	.221	*.942* ^*^	.792	.082	.971^**^
											*.954* ^*^	− .238	.597	*.982* ^**^	− .603	− .436	− .609
TMB											1	− .351	.526	.981^**^	.854	.213	.938^*^
												− .079	.720	.983^**^	− .336	− .148	− .343
ETN												1	− .108	− .202	.123	− .670	− .277
													− .083	− .173	.462	.529	.478
XYL													1	.432	.331	.445	.295
														.607	.024	.267	.025
CYM														1	.924^*^	.048	.962^**^
															− .460	− .302	− .469
DMS															1	− .082	*.827*
																.961^**^	*1.000* ^**^
PIR																1	− .086
																	.964^**^
DES																	1

Italicized entries show the strongest correlation between chemical compounds

*ACN* acetonitrile, *ACA* acetaldehyde, *MTH* methanethiol, *ACT* acetone, *PRO* 1-propanol, *ETH* ethanethiol, *BEN* benzene, *TOL* toluene, *BZA* benzaldehyde, *PIN* α-pinene, *TMB* 1,2,3-trimethylbenzene, *ETN* ethanol, *XYL* xylene, *CYM* cymene, *DMS* dimethyl sulphide, *PIR* pyridine, *DES* sulphide diethyl

^*^P < 0.05 (2-tailed)

^**^P < 0.01 (2-tailed)

Obtained information about high values of correlation coefficients indicates similar character of concentration changes of particular substances from the organosulphur compounds group due to sludge aging. Similar dependence was also observed in case of the investigations concerning emission of odorous compounds from 9 different regions of a landfill (Fang et al. 2012). A strong correlation within investigated groups of compounds, also including organosulphur ones, was observed based on analysis of linearity of identified compounds behavior. Additionally, a correlation between the investigated groups of compounds was evident, which could be a result of transformations of particular groups of compounds into the other ones.

In the case of aromatic hydrocarbons, a positive correlation was observed for both sludge samples, but only between a mixture of xylenes and benzaldehyde (*r*^2^_I_ = 1000 and *r*^2^_II_ = 0.979). For the remaining groups of compounds, only single, strong correlations were noticed between concentrations of the compounds belonging to the same chemical group. That is why, it is difficult to state, based on the investigations presented in this paper, that changes of concentration of the compounds, except of organosulphur ones, are correlated in a linear way. Obviously, one has to remember about small number of the investigated compounds, however they play an important role from the point of view of odor nuisance associated with processing of sludge cakes.

## Conclusions

The paper presents the attempt to apply the flux hood chamber coupled with proton transfer reaction–mass spectrometry technique for periodical monitoring of concentration changes of selected odorous compounds released during aging of stabilized and dewatered sludge cakes originating from two wastewater treatment plants. Combination of these solutions allowed determination of concentration changes of 17 odorous compounds released from the sludge cakes. Based on literature values of olfactory thresholds of the investigated compounds, it was revealed that organosulphur compounds (independently on the progress of sludge aging) had the biggest contribution to an increase in odor intensity connected with sludge cakes processing, in spite of the fact that their concentrations are lower than the ones of the other substances emitted from the sludge cakes. It was shown that even after 21 days of sludge aging, the sum of theoretical odor concentrations of monitored compounds still possessed significant load of odorous contaminants; the level of evaluated concentration was ca. 100 ou/m^3^. Moreover, it was found, comparing obtained results with literature data, that the results acquired with various types of chambers for measurement of volatile odorous compounds emission can differ between each other. These differences can result from flow rate of gas supplied to the chamber or construction design of the chamber, for example, implementation of internal mixing. It was also shown that in the case of organosulphur compounds, one could see correlations between concentrations of particular compounds belonging to this group.

Presented methodology of measurement of volatile odorous compounds employing the flux hood chamber coupled with PTR-TOF-MS technique allows monitoring of concentration of these compounds in real time. It enables collection of big amount of data without a need of time-consuming and expensive operations connected with preparation for analysis. So far, the PTR-MS technique has not been employed to monitoring of dewatered sludge. As compared to the GC-MS technique with thermal desorption, it is characterized by significantly shorter time of a single analysis and no need for sample preparation prior to analysis, which is in accordance with the green chemistry principles. Moreover, it excludes application of various types of sorbents for analytes sampling, which obviously influences on the final results of quantitative measurements (elimination of additional measurement errors). It seems that among other instrumental techniques, the PTR-MS can be one of the most effective approaches to real-time monitoring of concentration changes of particular odorous compounds. Nevertheless, it must be emphasized that dynamic olfactometry technique is the reference method, which provides holistic measurement of the entire mixture, not of its particular components, and takes into account odor interactions, for example, synergism.

The relatively high OAV of organosulfur compounds emitted from the sludge cakes cause an issue with the further utilization of the stabilized sludge. One could assume that after the unit processes depicted in Fig. 1, the content of these compounds ought to be characterized by significantly lower values of OAV. However, since their thermal utilization leads to the further emission of pollutants into the atmosphere in the form of sulfur dioxide, the improvement of the methane fermentation process through the disintegration of concentrated sludge seems to be a valid approach. The optimization of the parameters of these unit operations will result in a lower concentration of organosulfur compounds in the stabilized sludge, thus reducing the emission of odors.

## References

[CR1] Adams GA, Witherspoon J (2003) Identifying and controlling odor in the municipal wastewater environment phase II: impacts of in-plant parameters on biosolids odor quality. Report of Water Environment Research Foundation, Alexandria

[CR2] Alvarez FR, Shaul GM, Krishnan ER, Perrin DL, Rahman M (1999). Fate of terpene compounds in activated sludge wastewater treatment systems. J Air Waste Manage Assoc.

[CR3] Ammann C, Spirig C, Neftel A, Steinbacher M, Komenda M, Schaub A (2004). Application of PTR-MS for measurements of biogenic VOC in a deciduous forest. Int J Mass Spectrom.

[CR4] Biasioli F, Gasperi F, Odorizzi G, Aprea E, Mott D, Marini F, Autiero G, Rotondo G, Märk TD (2004). PTR-MS monitoring of odour emissions from composting plants. Int J Mass Spectrom.

[CR5] Blake RS, Monks PS, Ellis AM (2009). Proton-transfer reaction mass spectrometry. Chem Rev.

[CR6] Bokowa AH (2010). The review of the odour legislation. Proc Water Environ Fed.

[CR7] Bruno P, Caselli M, de Gennaro G, Solito M, Tutino M (2007). Monitoring of odor compounds produced by solid waste treatment plants with diffusive samplers. Waste Manag.

[CR8] Burlingame GA, Suffet IH, Khiari D, Bruchet AL (2004). Development of an odor wheel classification scheme for wastewater. Water Sci Technol.

[CR9] Byliński H, Gębicki J, Dymerski T, Namieśnik J (2017). Direct analysis of samples of various origin and composition using specific types of mass spectrometry. Crit Rev Anal Chem.

[CR10] Byliński H, Kolasińska P, Dymerski T (2017). Determination of odour concentration by TD-GC×GC–TOF-MS and field olfactometry techniques. Monatsh Chem.

[CR11] Byliński H, Dymerski T, Gębicki J, Namieśnik J (2018). Complementary use of GCxGC–TOF–MS and statistics for differentiation of variety in biosolid samples. Monatsh Chem.

[CR12] Capelli L, Sironi S, del Rosso R (2013). Odor sampling: techniques and strategies for the estimation of odor emission rates from different source types. Sensors (Switzerland).

[CR13] Capelli L, Sironi S, Del Rosso R, Guillot JM (2013). Measuring odours in the environment vs. dispersion modelling: a review. Atmos Environ.

[CR14] Cappellin L, Karl T, Probst M, Ismailova O, Winkler PM, Soukoulis C, Aprea E, Märk TD, Gasperi F, Biasioli F (2012). On quantitative determination of volatile organic compound concentrations using proton transfer reaction time-of-flight mass spectrometry. Environ Sci Technol.

[CR15] Carrera-Chapela F, Donoso-Bravo A, Souto JA, Ruiz-Filippi G (2014). Modeling the odor generation in WWTP: an integrated approach review. Water Air Soil Pollut.

[CR16] Carrera-Chapela F, Donoso-bravo A, González JA (2016). Air emissions from a sludge thickener: dynamic data for air quality models. Chem Eng Trans.

[CR17] Carrera-Chapela F, Donoso-Bravo A, Jeison D, Díaz I, Gonzalez JA, Ruiz-Filippi G (2016). Development, identification and validation of a mathematical model of anaerobic digestion of sewage sludge focusing on H_2_S formation and transfer. Biochem Eng J.

[CR18] Chen YC, Higgins MJ, Beightol SM, Murthy SN, Toffey WE (2011). Anaerobically digested biosolids odor generation and pathogen indicator regrowth after dewatering. Water Res.

[CR19] Chen WH, Lin SJ, Lee FC, Chen MH, Yeh TY, Kao CM (2017). Comparing volatile organic compound emissions during equalization in wastewater treatment between the flux-chamber and mass-transfer methods. Process Saf Environ Prot.

[CR20] Cieślik B, Konieczka P (2016). Sewage sludge management methods. Challenges and opportunities. Arch Waste Manage Environ Prot.

[CR21] Cui L, Zhang Z, Huang Y, Lee SC, Blake DR, Ho KF, Wang B, Gao Y, Wang XM, Louie PKK (2016). Measuring OVOCs and VOCs by PTR-MS in an urban roadside microenvironment of Hong Kong: relative humidity and temperature dependence, and field intercomparisons. Atmos Meas Tech.

[CR22] Fang JJ, Yang N, Cen DY (2012). Odor compounds from different sources of landfill: characterization and source identification. Waste Manag.

[CR23] Fisher RM, Barczak R, Alvarez Gaitan J (2017). Odorous volatile organic compound ( VOC ) emissions from ageing anaerobically stabilised biosolids. Water Sci Technol.

[CR24] Fisher RM, Le-Minh N, Sivret EC (2017). Distribution and sensorial relevance of volatile organic compounds emitted throughout wastewater biosolids processing. Sci Total Environ.

[CR25] Fisher RM, Barczak RJ, Suffet IHM (2018). Framework for the use of odour wheels to manage odours throughout wastewater biosolids processing. Sci Total Environ.

[CR26] Fisher RM, Le-Minh N, Alvarez-Gaitan JP (2018). Emissions of volatile sulfur compounds (VSCs) throughout wastewater biosolids processing. Sci Total Environ.

[CR27] Fisher RM, Shammay A, Alvarez-Gaitan JP, Stuetz RM (2018). Sewer catchment effects on wastewater and biosolids odour management. Water Sci Technol.

[CR28] Gębicki J (2016). Application of electrochemical sensors and sensor matrixes for measurement of odorous chemical compounds. Trends Anal Chem.

[CR29] Gębicki J, Byliński H, Namieśnik J (2016). Measurement techniques for assessing the olfactory impact of municipal sewage treatment plants. Environ Monit Assess.

[CR30] Gruchlik Y, Heitz A, Joll C, Driessen H, Fouché L, Penney N, Charrois JWA (2013). Odour reduction strategies for biosolids produced from a Western Australian wastewater treatment plant: results from phase I laboratory trials. Water Sci Technol.

[CR31] Guillot J, Milan B (2016). E-noses: actual limitations and perspectives for environmental odour analysis. Chem Eng Trans.

[CR32] Guillot J, Clincke A, Guilleman M (2014). Odour emission from liquid and solid area sources : a large intercomparison of sampling devices. Chem Eng Trans.

[CR33] Hewitt CN, Hayward S, Tani A (2003). The application of proton transfer reaction-mass spectrometry (PTR-MS) to the monitoring and analysis of volatile organic compounds in the atmosphere. J Environ Monit.

[CR34] Hudson N, Gies A, Duperouzel D (2006). Assessment of permeable covers for odour reduction in piggery effluent ponds. 2. Field-scale trials. Bioresour Technol.

[CR35] Hudson N, Ayoko GA, Collman G (2008). Long-term assessment of efficacy of permeable pond covers for odour reduction. Bioresour Technol.

[CR36] Hudson N, Ayoko GA, Dunlop M, Duperouzel D, Burrell D, Bell K, Gallagher E, Nicholas P, Heinrich N (2009). Comparison of odour emission rates measured from various sources using two sampling devices. Bioresour Technol.

[CR37] Jordan A, Haidacher S, Hanel G, Hartungen E, Herbig J, Märk L, Schottkowsky R, Seehauser H, Sulzer P, Märk TD (2009). An online ultra-high sensitivity proton-transfer-reaction mass-spectrometer combined with switchable reagent ion capability (PTR+SRI−MS). Int J Mass Spectrom.

[CR38] Kim M, Chowdhury MMI, Nakhla G, Keleman M (2017). Synergism of co-digestion of food wastes with municipal wastewater treatment biosolids. Waste Manag.

[CR39] Klenbusch MR (1986) Measurement of gaseous emission rates from land surfaces using an emission isolation flux chamber. User’s Guid EPA-600/8-86/008 US Environ Prot Agency, Washington, DC

[CR40] Konieczka P, Namieśnik J (2007). Ocena i kontrola jakości wyników pomiarów analitycznych.

[CR41] Kosek K, Kozak K, Kozioł K (2018). The interaction between bacterial abundance and selected pollutants concentration levels in an arctic catchment (Southwest Spitsbergen, Svalbard). Sci Total Environ.

[CR42] Lewkowska P, Cieślik B, Dymerski T, Konieczka P, Namieśnik J (2016). Characteristics of odors emitted from municipal wastewater treatment plant and methods for their identification and deodorization techniques. Environ Res.

[CR43] Leyris C, Guillot JM, Fanlo JL, Pourtier L (2005). Comparison and development of dynamic flux chambers to determine odorous compound emission rates from area sources. Chemosphere.

[CR44] Lindinger W, Hansel A, Jordan A (1998). On-line monitoring of volatile organic compounds at pptv levels by means of proton-transfer-reaction mass spectrometry (PTR-MS) medical applications, food control and environmental research. Int J Mass Spectrom Ion Process.

[CR45] Lindinger W, Fall R, Karl TG (2001). Environmental, food and medical applications of proton-transfer-reaction mass spectrometry (PTR-MS). Adv Gas Phase Ion Chem.

[CR46] Lucernoni F, Capelli L, Sironi S (2016). Comparison of different approaches for the estimation of odour emissions from landfill surfaces. Waste Manag.

[CR47] Lucernoni F, Tapparo F, Capelli L, Sironi S (2016). Evaluation of an Odour Emission Factor (OEF) to estimate odour emissions from landfill surfaces. Atmos Environ.

[CR48] Mrowiec B, Suschka J, Keener TC (2005). Formation and biodegradation of toluene in the anaerobic sludge digestion process. Water Environ Res.

[CR49] Nagata Y (2003) Measurement of odor threshold by triangle odor bag method. Odor Meas Rev:118–127

[CR50] Nicolas J, Craffe F, Romain AC (2006). Estimation of odor emission rate from landfill areas using the sniffing team method. Waste Manag.

[CR51] Novak JT, Sadler ME, Murthy SN (2003). Mechanisms of floc destruction during anaerobic and aerobic digestion and the effect on conditioning and dewatering of biosolids. Water Res.

[CR52] Parker D, Ham J, Woodbury B, Cai L, Spiehs M, Rhoades M, Trabue S, Casey K, Todd R, Cole A (2013). Standardization of flux chamber and wind tunnel flux measurements for quantifying volatile organic compound and ammonia emissions from area sources at animal feeding operations. Atmos Environ.

[CR53] Qi Y, Dentel SK, Herson DS (2008). Effect of total solids on fecal coliform regrowth in anaerobically digested biosolids. Water Res.

[CR54] Rinne J, Taipale R, Markkanen T, Ruuskanen TM, Hellén H, Kajos MK, Vesala T, Kulmala M (2007). Hydrocarbon fluxes above a Scots pine forest canopy: measurements and modeling. Atmos Chem Phys.

[CR55] Rosenfeld PE, Henry CL, Dills RL, Harrison RB (2001). Comparison of odor emissions from three different biosolids applied to forest soil. Water Air Soil Pollut.

[CR56] Roy MM, Dutta A, Corscadden K (2011). Review of biosolids management options and co-incineration of a biosolid-derived fuel. Waste Manag.

[CR57] Sharma B, Sarkar A, Singh P, Singh RP (2017). Agricultural utilization of biosolids: a review on potential effects on soil and plant grown. Waste Manag.

[CR58] Sironi S, Capelli L, Céntola P, del Rosso R, Il Grande M (2005). Odour emission factors for assessment and prediction of Italian MSW landfills odour impact. Atmos Environ.

[CR59] Taipale R, Ruuskanen TM, Rinne J, Kajos MK, Hakola H, Pohja T, Kulmala M (2008). Technical note: quantitative long-term measurements of VOC concentrations by PTR-MS measurement, calibration, and volume mixing ratio calculation methods. Atmos Chem Phys Discuss.

[CR60] Tani A, Kato S, Kajii Y, Wilkinson M, Owen S, Hewitt N (2007). A proton transfer reaction mass spectrometry based system for determining plant uptake of volatile organic compounds. Atmos Environ.

[CR61] Van Harreveld T, Guillot J, Baas N et al (2013) Progress of EN13725 revision, the standard used to determine odour concentration and odour treatment efficiency. In: Proceedings of the Biotechniques for Air Pollution Control and Bioenergy, Nimes, France

[CR62] Woźniak MK, Wiergowski M, Aszyk J, Kubica P, Namieśnik J, Biziuk M (2018). Application of gas chromatography–tandem mass spectrometry for the determination of amphetamine-type stimulants in blood and urine. J Pharm Biomed Anal.

[CR63] Wu C, Liu J, Zhao P, Li W, Yan L, Piringer M, Schauberger G (2017). Evaluation of the chemical composition and correlation between the calculated and measurement odour concentration of odorous gases from a landfill in Beijing, China. Atmos Environ.

[CR64] Xu C, Chen W, Hong J (2014). Life-cycle environmental and economic assessment of sewage sludge treatment in China. J Clean Prod.

[CR65] Zhao J, Zhang R (2004). Proton transfer reaction rate constants between hydronium ion (H_3_O^+^) and volatile organic compounds. Atmos Environ.

